# Calibration and validation of solar radiation-based equations to estimate crop evapotranspiration in a semi-arid climate

**DOI:** 10.1007/s00484-023-02566-5

**Published:** 2023-11-27

**Authors:** Georgios Nikolaou, Damianos Neocleous, Antonio Manes, Evangelini Kitta

**Affiliations:** 1 Department of Agriculture Crop Production and Rural Environment, School of Agricultural Sciences, University of Thessaly, Fytokou Str., 38446 Volos, Greece; 2 Department of Natural Resources and Environment, Ministry of Agriculture, Agricultural Research Institute, 1516 Nicosia, Cyprus; 3 Netsens Srl, 50041 Calenzano, (FI) Italy

**Keywords:** Abtew equation, Citrus, Extraterrestrial radiation, Irrigation scheduling, Jensen-Haise equation, Mediterranean region, Olives

## Abstract

The Abtew and Jensen-Haise solar radiation-based equations were used to estimate evapotranspiration, considering the limited climatic data in many locations. Both equations were proven to successfully predict the potential evapotranspiration (ET_O_) compared with the standard Penman–Monteith (PM) method in two Mediterranean countries. Calibration of the constant coefficient* k* of the Abtew equation showed substantial differences compared to recommended values (1.22 vs. 0.53), with the highest values observed during September (1.46). Validation of ET_O_ measurements using calibrated Abtew equation against the PM method indicated a high correlation coefficient (r^2^ = 0.97, RMSE = 0.61). Further, evapotranspiration requirements, using the calibrated empirical equation, were calculated for olives (449 mm) and citrus (807 mm) showing a good agreement with recommended values for dry climate regions. Therefore, the tested equations could be safely used to predict frequencies and doses of irrigation in semi-arid climates, considering limited climatic data availability.

## Introduction

The water consumption of a crop (i.e., Evapotranspiration-ET_C_-; soil evaporation-E, and plant transpiration-T) depends mainly on a daily phenomenon during which a transition occurs from the liquid phase of water to the vapour phase. It causes a reduction in plants’ temperature and requires a certain amount of energy, which is provided by the available radiative and convective energy at the plant and soil evaporating surfaces. This available energy represents the crop water demand, ET_C_ (Kittas 1990). In arid and semi-arid climates, agriculture is closely related to the rate of evapotranspiration and the probability of precipitation. For example, under arid conditions, 95% of the annual precipitation is estimated to be consumed by ET_C_ (Melesse et al. 2009). Thus, considering the water and energy crises, the need for information on evapotranspiration has become more important today than a few decades ago. Frequently in agriculture practice, we estimate ET_C_ by multiplying potential evapotranspiration (i.e., ET_O_; the capacity of the atmosphere to remove water from a surface over a specified region) from a reference canopy such as the turf grass under a non-limiting water supply with a “crop coefficient value”, which characterizes each crop and is related to its developmental stage (Nikolaou et al. 2022). However, experimental determination of ET_O_ is only possible at very limited sites because of its complexity and the high cost of equipment required (Flores-Velazquez et al. 2022).

Today, ET_O_ could be estimated based on real-time climatic data (i.e., wind speed, relative humidity, air temperature, solar radiation, and sunshine hours) recorded by automated agro-meteorological climatic stations (Shirmohammadi-Aliakbarkhani and Saberali 2020). The Penman–Monteith (PM) method, released by the Food and Agriculture Organization (FAO), represents an internationally recommended model since 1990 that could be used for evapotranspiration estimation (Allen et al. 2006; Achparaki et al. 2012; Wang et al. 2021). However, in many locations, the complete dataset of meteorological variables required for the PM method is not available (Bogawski and Bednorz 2014). Thus to overcome this problem, several scientists and researchers from around the world applied a variety of empirical equations to calculate evapotranspiration (such as the Hargraves, the Blanley Criddle, the Thornthwaite, and the Makkink), but none of them could be deemed flawless due to the vast variations in climatic conditions in different parts of the world (Yates and Strzepe 1994; Faruk Bin Poyen et al. 2016). However, the strong dependence of evaporation on the radiation energy term has been generally accepted (Islam and Rashidul Alam 2021; Flores-Velazquez et al. 2022). Evapotranspiration estimation based on solar radiation measurements eliminates the effect of the surface albedo and minimizes the contribution of the aerodynamic term. Xu and Singh (2000) found that under clear sky conditions, there is a great balance between evapotranspiration accuracy estimation, simplicity of the solar radiation method, and robustness. However, under low solar radiation and cloudiness, evapotranspiration underestimation was a common problem since only an amount between 70 and 75% of the daily ET_C_ variance is explained by solar radiation alone (Melesse et al. 2009). In another case, for soilless-based greenhouse crops the accumulated solar-radiation method has been applied for a long time, matching the diurnal evapotranspiration fluctuation with solar radiation as a sustainable water-saving approach (Katsoulas et al. 2006; Nikolaou et al. 2017). However, the main disadvantage of using equations with very few input data is the performance efficiency under different climate systems as mentioned elsewhere (Aschale et al. 2022).

Among radiation-based methods, for warm climates, the Jensen-Haise method has a very good performance rating for ET_O_ calculation (Shirmohammadi-Aliakbarkhani and Saberali 2020; Gharehbaghi and Kaya 2022). The Jensen-Haise was initially calibrated under semi-arid conditions using solar radiation and air temperature (Jensen and Haise 1963). The primary gain in Jensen-Haise accuracy comes from the inclusion of air temperature into the ET_O_ equation. The argument to use temperature is that both components of evaporation in the PM equation are related to the air temperature (Yates and Strzepe 1994). Abtew is another simple method that could be applied for ET_O_ calculations where the only available climatic data is solar radiation (Mengistu and Amente 2017; Islam and Rashidul Alam 2021). It was originally developed for warm and humid environments; therefore, recalibration under arid and semi-arid conditions is recommended to increase the model’s accuracy ( Xu and Singh 2000; Samaras et al. 2014; Mengistu and Amente 2017). Considering the effectiveness of the Abtew equation, the *k* coefficient represents the relation between solar radiation and water consumption in the reference canopy used for the determination of the potential evapotranspiration. Initially, a constant *k* value of 0.53 was proposed. Working within greenhouses (Kittas 1990) showed that *k* values ranged from 0.44 to 0.52, increasing up to 0.67 in a semi-arid environment.

Irrigation scheduling is not widely used in many parts of the world, despite the abundance of evapotranspiration estimation-based decision support systems that have been created over the past few decades (Giannakis et al. 2016; Taghvaeian et al. 2020). Therefore, the adaptability of simpler methods for use in predicting when and how much water is required for any particular irrigation scheme is worth investigating, especially in geographical areas where there is limited climate monitoring (Melesse et al. 2009).Therefore, in the present manuscript, we focus on two empirically based ET_O_ equations that use limited and easily recorded climatic data (i.e., solar radiation and air temperature). Given that irrigation water is available, a wide variety of crops can be grown in the Mediterranean because of its temperate climate. For example, in arid and semi-arid regions, irrigated open-field crops are usually cultivated from March to September, in most cases under completely clear sky conditions. The total irrigation requirements for the main crops may rise from 350 (e.g., vegetables) to 2400 (e.g., colocasia) mm (i.e., 3500–24000 cubic meters of water per hectare) (Dalias et al. 2019; Nikolaou et al. 2020a, b). Because crops in these areas depend on sufficient supplies of high-quality water, the availability of water has always been an issue (Sánchez-Molina et al. 2015; Nikolaou et al. 2020a, b). Particularly, olive crops (Olea europaea L.) are considered one of the most economically and ecologically important tree crops in the Mediterranean area (Sofoulaki et al. 2023). Olive is resistant to aridity; however, the higher frequency and severity of droughts in the future would result in an average increase of 18.5% of the irrigation demand over the Mediterranean (Fraga et al. 2021). On the other hand, there has been a tendency over the last decade toward high plant olive crop density (up to 2500 plants ha^−1^ as opposed to the current practice of 350 plants ha^−1^) as it has been reported to be a good strategy in terms of orchard productivity under semi-arid conditions (Egea et al. 2017).

Citrus is another crop widespread worldwide, with the countries around the Mediterranean basin constituting important producers (Sofoulaki et al. 2023). However, in semi-arid conditions, citrus evapotranspiration falls in the conventional range of 700–1300 (mm), with an average of 1000 (mm) during the irrigation period (Abou Ali et al. 2023). Thus, it is important to determine the citrus water requirements by designing simple, practical, and precise methods to optimize irrigation and to adopt precise irrigation scheduling and management techniques aiming for water savings (Puig-Sirera et al. 2021).

In view of the above, in this study, climatic data from two semi-arid Mediterranean regions, Cyprus and Italy, were utilized to compare the performances of the Abtew and Jensen-Haise radiation-based equations with the Penman–Monteith equation for calculating potential evapotranspiration (ET_O_). A modified Abtew equation based on extraterrestrial solar radiation is also tested for citrus and olive crop ET_C_ calculation as an ex-ante irrigation scheduling tool.

## Materials and methods

### Experimental setup

For this study, three agro-meteorological stations were chosen, two of which were located in Cyprus and one in Italy, all of which were in semi-arid Mediterranean environments.

Station A: Inland area of southern Cyprus, in aromatic plants, lat. 35° 08′ 8.70 N, long. 33° 24′ 9.60" Ε, altitude 165 m.s.l., with a hot semi-arid climate (hot, sometimes extremely hot, summers and warm to cool winters), classified as BSh by the Köppen-Geiger system.

Station B: Mountainous location of southern Cyprus, in olive plants, lat. 34° 57′ 17.00 N, long. 33° 23′ 24.00" Ε, lat. 330 m.s.l., with a hot Mediterranean climate (hot dry- summers and mild, wet winters), classified as Csa by the Köppen-Geiger system.

Station C: Coastal area of northern Italy, in vegetable plants, lat. 41° 10′ 25.06 N, long. 16° 36′ 30.78" Ε, 60 m.s.l., with a hot Mediterranean climate, classified as Csa by the Köppen-Geiger system.

The selected study locations are characterized by the typical climates of the Mediterranean area, with mild winters (annual rainfall of about 320–340 mm between October and March), warm summers, and an average annual sunshine duration of about 3 332 h. The summer months are mostly dry, with relatively high global and horizontal beam radiation intensities. The Mediterranean climate means that evaporation rates are quite high but also reasonably constant from year to year. Historic data allows accurate predictions of evaporation losses, but such losses are considered inevitable (Cox 1999).

**Fig. 1:**
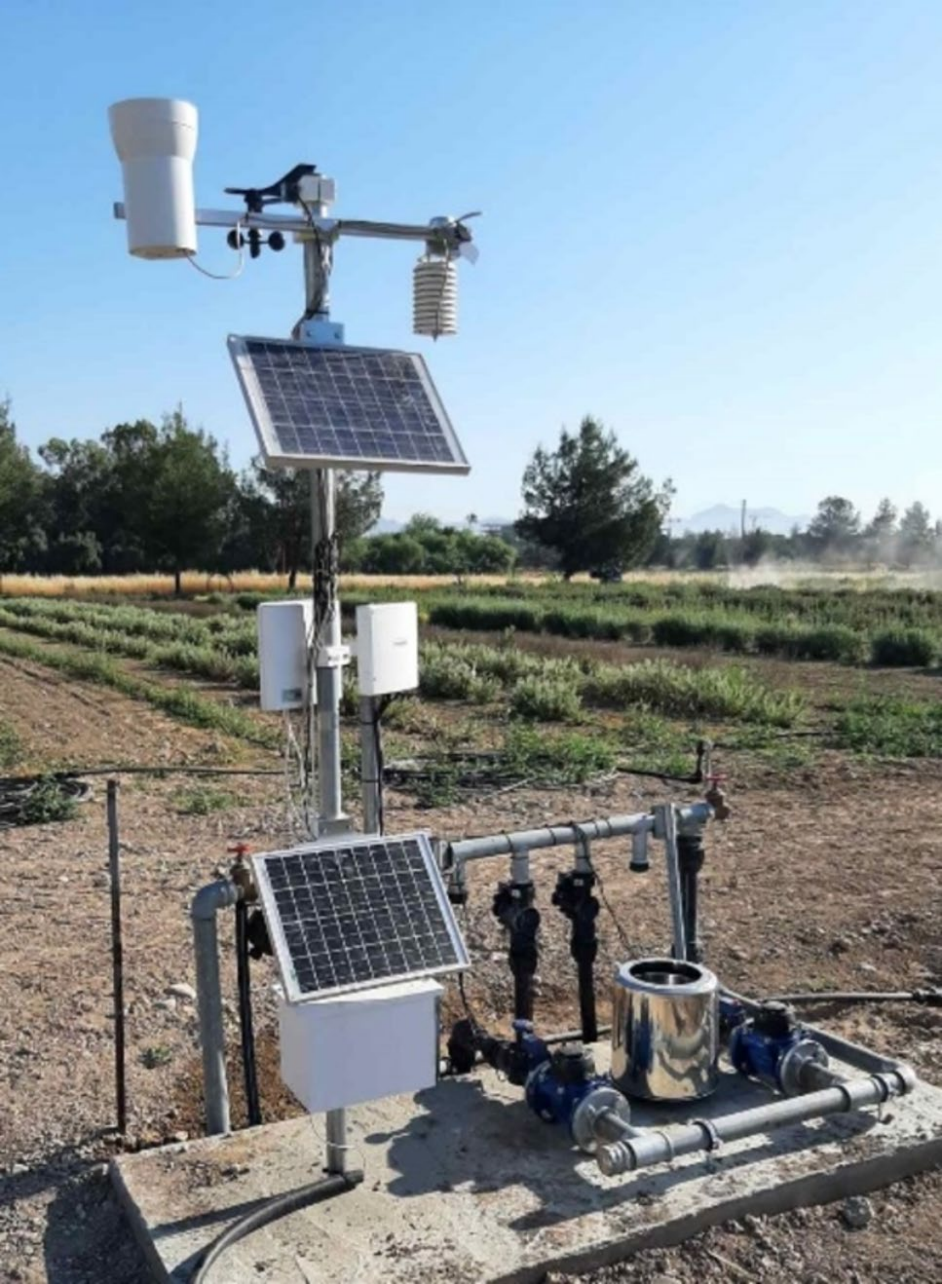
Station A located in an inland area of southern Cyprus

In each study location, an automatic agro-meteorological climatic station (MeteoSense 4.0; Netsens; Calenzano; Florence; Italy), with sensors mounted on a pole at 2 m height (Fig. 1). It consisted of a wind sensor (1–67 m s^−1^, accuracy 5%; direction 0–360^◦^, accuracy 7^◦^), a rain collector (tipping bucket, resolution 0.2 mm), a solar radiation sensor (0–1800 W m^−2^, accuracy 5%), a thermo-hydrometer for air temperature (-25 to + 85 °C; accuracy 0.5 °C), and air humidity (0–100% RH; accuracy 3%). A solar panel is used as a source of power, integrated with a storage battery. Climatic data were forwarded in real-time to the Netsens LiveData platform (data cloud platform; IoT system) with the use of a SIM card. Climatic data were recorded at 30-s intervals and transmitted as average data every 5 min, while on the cloud platform, data can be downloaded on an hourly basis.

### Theoretical approach

#### Penman–Monteith method

As a result of an expert consultation held in May 1990, the FAO Penman–Monteith method is recommended as the sole standard equation for the definition and computation of the reference evapotranspiration (Allen et al. 1998).

1 $${ET}_{OPM}= \frac{0.408\Delta \left({R}_{N}-G\right)+\gamma \frac{900}{{\rm T}+273}{u}_{2}({e}_{s}-{e}_{a})}{\Delta +\gamma (1+0.34{u}_{2})}$$

Where ET_OPM_ is the estimated reference evapotranspiration value (mm d^−1^), R_N_ is the net radiation at the crop surface (MJ m^−2^ day^−1^), G is the soil heat flux density (MJ m^−2^ day^−1^), T is the mean daily air temperature at 2 m height (°C), u_2_ is the wind speed at 2 m height (m s^−1^), e_s_ is the saturation vapour pressure (kPa), e_a_ is the actual vapour pressure (kPa), e_s_-e_a_ is the saturation vapor pressure deficit (kPa °C), Δ is the slope of the vapour pressure curve (kPa °C^−1^), γ is the psychrometric constant (kPa °C^−1^).

#### Jensen and Haise method

Jensen and Haise (1963) proposed an empirical equation for semi-arid and arid regions that estimated potential evapotranspiration based on solar radiation and air temperature as follows:

2 $${ET}_{OJH}= {R}_{S}\left(0.025 {T}_{a}+0.08\right)$$

Where ET_ΟJH_ is the estimated reference evapotranspiration value (mm d^−1^), T_α_ the mean air temperature (°C d^−1^), R_S_ is the mean solar radiation (MJ m^−2^ d^−1^). The air temperature indirectly introduced the contribution of the aerodynamic term into the estimate of ET_O_. The equation, which is referred to as a radiation-based model, has been derived from data collected in arid regions of the western part of the United States and should yield satisfactory results in areas with similar climates (Jensen and Haise 1963).

#### Abtew method

To account for cases where the only available climatic data is solar radiation, the Abtew equation is recommended (Abtew 1996):

3 $${ET}_{OA}= k \frac{{R}_{S}}{\lambda }$$

Where ET_ΟΑ_ is the estimated reference evapotranspiration value (mm d^−1^), *k* represents a dimensionless coefficient, R_S_ is the mean solar radiation (MJ m^−2^ d^−1^); λ is the latent heat vaporization (2.45 MJ kg^−1^ according to Allen et al. 2006). The Abtew equation was originally developed and used in warm and humid wetland environments (Samaras et al. 2014). Therefore, we proceeded with the calibration of the Abtew *k* coefficient for semi-arid environments by rewriting Eq. 3 as:

4 $$\mathrm{k}= \frac{\lambda {ET}_{OPM}}{{R}_{S}}$$

For the calculation of potential evapotranspiration in mm d^−1^, the solar radiation (R_S_) must be converted to mm d^−1^ (Allen et al. 1998; Jaafar and Ahmad 2019; Mengistu and Amente 2017):

5 $${R}_{S}\left(mm {d}^{-1}\right)=\frac{{R}_{S}\left(MJ {m}^{-2 }{d}^{-1}\right)}{(\lambda *{\rho }_{W})}$$

Where ρ_w_ is the density of water (1.000 kg m^−3^).

#### *Estimating crop evapotranspiration (*ETc*)*

In the single crop coefficient approach, the crop coefficient (K_C_) integrates differences in crop transpiration rates and soil water evaporation between the crop and the grass reference surface. The Kc is the ratio of ETc to ETo; it represents an integration of the effects that distinguish a crop from the reference grass (Xiang et al. 2020). In the crop coefficient method, evapotranspiration (ET_C_) is given below (Simbeye et al. 2023):

6 $${ET}_{C}= {\mathrm{K}}_{C}*{ET}_{O}$$

Where ET_C_ is the estimated crop evapotranspiration value (mm d^−1^), K_C_ is a crop coefficient (dimensionless; varies with the growth stage of the crop), and ET_O_ is a reference crop evapotranspiration (mm d^−1^).

#### *Estimating *ETc* using a modified Abtew equation*

The earth’s solar radiation fluxes (R_S_) can be estimated under clear sky conditions based on extraterrestrial (R_A_) radiation (Appendix Table 4) and station elevation above sea level, following Allen, (1998) transformation equation (R_S_ = 0.75 + 2 10–^5^ ζ) R_A_/λ). The importance of estimating R_Ν_ based on R_A_ could be applied in cases of total absence of current measurements of radiation. The modified Abtew equation can be rewritten as follows:

7 $${ET}_{OA}= k \frac{\left(0.75+2 {10}^{-5} \zeta \right){R}_{A}}{\lambda }$$

Subsequently, crop evapotranspiration (ET_C_) can be estimated by substituting (7) in (6) using the following formula:

8 $${ET}_{C}= {\mathrm{K}}_{C}*k \frac{\left(0.75+2 {10}^{-5} \zeta \right){R}_{A}}{\lambda }$$

Where R_A_ is the extraterrestrial radiation (Mj m^−2^ d^−1^), ζ is the station elevation (m), K_C_ is the crop coefficient.

### Experimental methodology

Considering climatic data recorded in stations A and B (Cyprus) and calculated potential evapotranspiration (ET_OPM_; Penman–Monteith equation), we proceeded with calibration of the constant coefficient *k* of the Abtew equation. The model coefficient was then tested (ET_OA_) and compared with the ET_OPM_ in a different location and cropping conditions (station C; Italy). To check the suitability of the proposed modified Abtew equation (Eq. 8), the evapotranspiration requirements for two main Mediterranean crops (i.e., olives and citrus) were estimated based on extraterrestrial radiation.The Jensen-Haise solar radiation-based equation was also evaluated in comparison with the Penman–Monteith equation for calculating potential evapotranspiration (stations A, B, C), considering the limited climatic data in many locations.

Crop coefficients were used as proposed by Er-Raki et al. (2008) for olives (i.e., K_C_ ini = 0.65, K_C_ mid = 0.45, K_C_ late = 0.65) and by Jamshidi et al. (2020) for citrus (i.e., K_C_ = 0.71 to 0.96) for semi-arid regions. The olive season lasts in the Mediterranean regions from early March to November, and the lengths of the several crop development stages (L-ini, L-dev, L-mid, and L-late) are, respectively, 30, 90, 60, and 90 days. For citrus crops, the growing season typically starts in early February with flower bud induction, followed by flowering from mid-March to April. The length of crop development stages (L-ini, L-dev, L-mid, and L-late) is, respectively, 60, 90, 120, and 95 days (Allen et al. 1998; Fraga et al. 2021; Abou Ali et al. 2023).

The reliability of empirical equations for calculating ET_O_ increases when the methods are calibrated for each crop in each region. This is necessary because the effective rooting depth and the permissible water deficit for each crop, as well as soil–water retention characteristics, are factors that have to be taken into consideration. In addition, the considerable advection of energy from unirrigated surroundings (oasis effects) affects the capacity of the atmosphere to remove water from a surface over a specified region of each location.

### Statistical analysis

Climatic data were analyzed and comparisons of means were tested with ANOVA using a Statistical Package for the Social Sciences (IBM Corp. Release 2011. IBM SPSS Statistics for Windows, Version 20.0. Armonk, NY, USA: IBM Corp). Regression analysis was performed for the estimation of relationships between selected data.

## Results

### Climatic conditions

**Fig. 2:**
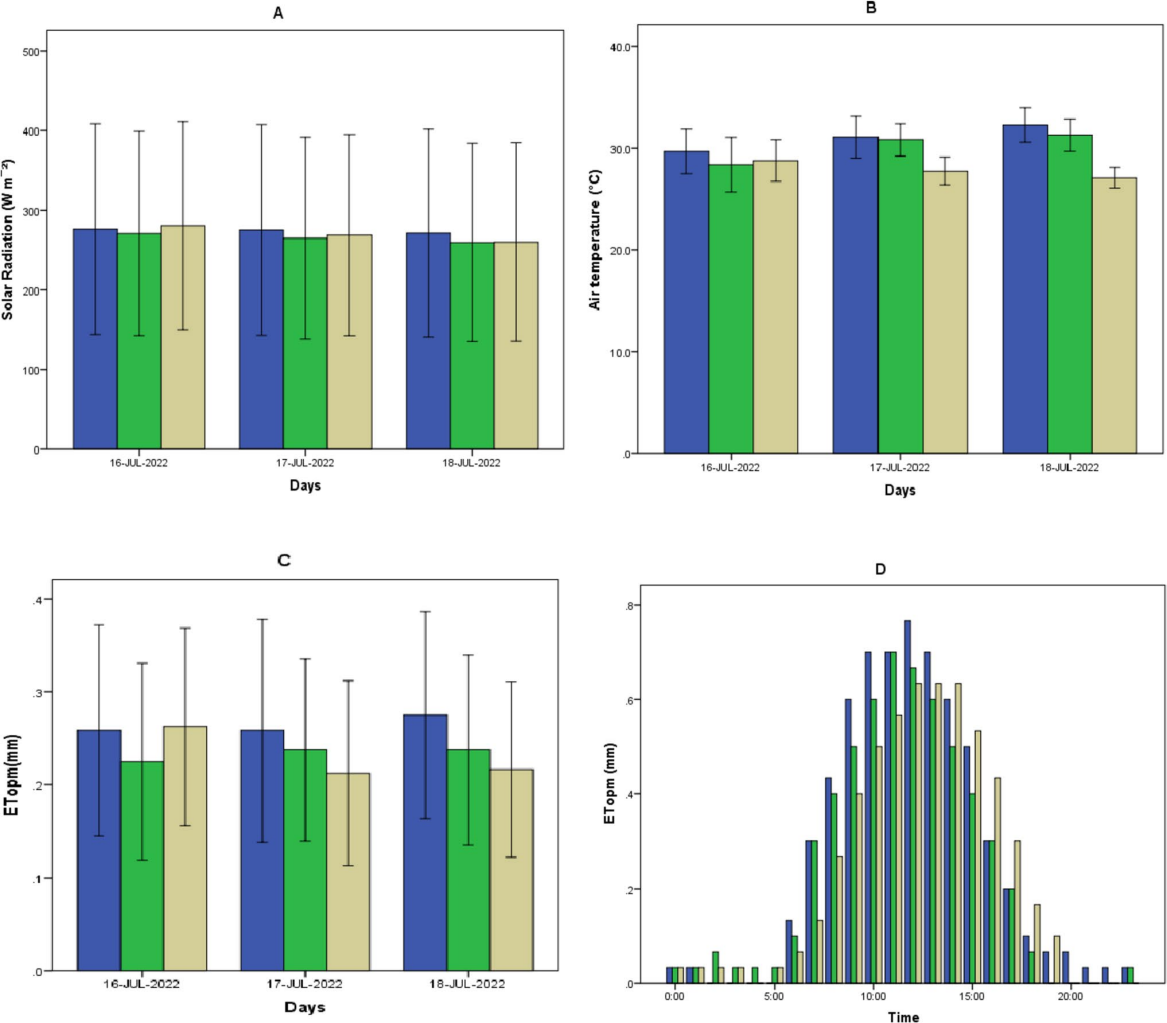
Daily solar radiation (A; mean hourly; W m^−2^), daily air temperature (B; mean hourly; °C), and potential evapotranspiration based on the Penman–Monteith equation (C; mean hourly; mm) in a 3-representative day period in July; ET_OPM_ in a 24-h period (D; mean hourly; mm); blue bars (agro-meteorological station A; altitude 165 m; Cyprus); green bars (agro-meteorological station B; altitude 330 m; Cyprus); brown bars (agro-meteorological station C; altitude 60 m; Italy)

Table 1 summarizes the mean values of air temperature, relative humidity, solar radiation, evapotranspiration (ET_OPM_; potential evapotranspiration; Penman–Monteith FAO-56; Eq. 1), and 10-day accumulated precipitation for each agro-meteorological station during daylight hours. The mean daily solar radiation values were slightly higher over the study period for station A at 448 W m^−2^ (maximum 869 W m^−2^) compared to station B at 442 W m^−2^ (maximum 866 W m^−2^) or C at 411 W m^−2^ (maximum 899 W m^−2^) with an exception during the second 10-day interval measurement period in July where station C recorded higher solar radiation (Table 1 and Fig. 2A). This was probably due to a Sahara dust transport to Cyprus, reducing the amount of solar radiation reaching the soil surface. The minimum daily sunshine hours were 5 in March and increased to 12 in July.

**Table 1:** Mean hourly values of air temperature, relative humidity, and solar radiation for daylight hours (> solar radiation 50 Wm^−2^), mean daily evapotranspiration, and 10-day accumulated precipitation in agro-meteorology stations. Values in parenthesis represent (± standard error)

	Station A						Station B					Station C				
Month	[1]	[2]	[3]	[4]	[5]	[6]*	[2]	[3]	[4]	[5]	[6]	[2]	[3]	[4]	[5]	[6]
March	1	14.7 (0.2)	60.3 (1.3)	0.04	311 (18.3)	1.7 (0.2)	n.a	n.a	n.a	n.a	1.7 (0.1)	8.6 (0.3)	60.4 (1.4)	7.8	232 (15.1)	1.5 (0.2)
	2	9.5 (0.2)	48.2 (1.7)	0.06	344 (18.7)	1.9 (0.2)	9.1 (0.4)	44.5 (1.8)	0.61	297 (26.2)	1.1 (0.2)	11.5 (0.3)	58.8 (1.2)	-	339 (16.5)	1.9 (0.1)
	3	14.8 (0.5)	47.8 (1.6)	0.01	373 (20.1)	2.0 (0.3)	13.8 (0.4)	46.3 (1.5)	1.06	333 (18.0)	1.7 (0.3)	14.8 (0.3)	58.7 (1.4)	0.94	317 (18.7)	2.0 (0.3)
April	1	22.8 (0.4)	42.0 (1.9)	-	419 (21.5)	3.8 (0.1)	23.0 (0.3)	35.1 (1.2)	1.06	433 (20.3)	3.4 (0.1)	14.6 (0.3)	59.5 (1.1)	2.0	302 (18.9)	2.2 (0.2)
	2	20.5 (0.3)	39.0 (1.4)	-	447 (22.2)	3.9 (0.1)	19.9 (0.3)	37.2 (1.2)	-	434 (21.2)	3.4 (0.2)	15.8 (0.2)	53.9 (0.9)	-	409 (20.9)	3.0 (0.1)
	3	25.9 (0.4)	32.4 (1.3)	-	481 (22.3)	4.7 (0.1)	25.2 (0.4	31.1 (1.0)	-	473 (21.7)	4.1 (0.1)	19.1 (0.2)	55.8 (1.0)	0.13	397 (21.0)	3.2 (0.2)
May	1	23.8 (0.3)	40.8 (1.5)	-	457 (22.0)	4.4 (0.3)	22.9 (0.3)	40.0 (1.4)	-	422 (20.1)	3.8 (0.3)	18.5 (0.2)	68.0 (1.2)	7.87	370 (20.7)	2.8 (0.3)
	2	25.0 (0.4)	41.9 (1.4)	-	420 (23.9)	4.3 (0.3)	24.4 (0.3)	40.2 (0.8)	0.07	425 (22.6)	3.8 (0.3)	23.3 (0.2)	54.6 (1.0)	-	484 (23.0)	4.3 (0.2)
	3	29.3 (0.4)	34.6 (1.3)	-	486 (22.0)	5.5 (0.2)	29.0 (0.4)	30.2 (0.9)	0.04	487 (21.3)	5.0 (0.1)	25.9 (0.3)	59.8 (0.9)	-	445 (20.7)	4.2 (0.2)
June	1	28.0 (0.4)	52.3 (1.5)	-	440 (21.7)	4.6 (0.2)	26.8 (0.3)	54.9 (1.3)	-	461 (22.3)	4.4 (0.2)	26.9 (0.3)	58.2 (1.1)	0.22	453 (23.4)	4.3 (0.3)
	2	29.2 (0.4)	48.5 (1.5)	43.1	454 (22.8)	4.8 (0.4)	28.4 (0.4)	45.8 (1.6)	0.24	439 (22.9)	4.5 (0.3)	26.2 (0.2)	56.0 (0.7)	-	482 (22.2)	5.2 (0.1)
	3	30.8 (0.3)	35.8 (1.1)	-	474 (22.6)	5.5 (0.2)	29.9 (0.3)	33.7 (0.9)	0.64	473 (21.6)	5.2 (0.2)	30.8 (0.2)	49.7 (0.8)	-	437 (20.9)	5.2 (0.1)
July	1	32.7 (0.4)	35.8 (1.4)	-	497 (23.6)	5.5 (0.4)	32.0 (0.3)	34.2 (1.2)		480 (21.80	5.5 (0.2)	29.0 (0.2)	56.4 (0.9)	1.27	470 (22.6)	5.1 (0.3)
	2	33.8 (0.3)	32.7 (0.9)	-	495 (22.7)	6.1 (0.1)	33.1 (0.3)	30.9 (0.9)	-	484 (21.70	5.4 (0.1)	28.4 (0.2)	49.9 (0.8)	-	504 (21.9)	5.3 (0.1)
	3	34.6 (0.3)	28.0 (1.0)	-	518 (21.7)	5.6 (0.6)	33.9 (0.2)	26.8 (0.6)	-	496 (20.7)	4.9 (0.5)	31.2 (0.2)	51.7 (0.8)	-	457 (21.4)	4.7 (0.5)
August	1	32.9 (0.3)	48.6 (1.3)	-	479 (21.4)	5.0 (0.1)	31.7 (0.3)	49.7 (1.2)	-	471 (20.8)	4.5 (0.1)	29.9 (0.1)	54.0 (0.6)	0.04	470 (20.8)	5.1 (0.1)
	2	32.5 (0.3)	45.6 (1.3)	-	475 (21.8)	5.0 (0.1)	31.7 (0.3)	44.4 (1.2)	-	438 (20.3)	4.3 (0.1)	28.6 (0.3)	56.7 (0.9)	0.87	384 (20.2)	3.8 (0.3)
	3	31.7 (0.3)	50.5 (1.4)	9.8	431 (20.5)	4.4 (0.2)	31.4 (0.3)	45.0 (1.0)	-	412 (19.50	3.9 (0.1)	27.0 (0.2)	62.3 (0.9)	0.28	403 (19.7)	3.7 (0.2)
September	1	31.9 (0.3)	41.3 (1.6)	-	453 (20.9)	4.5 0.2)	31.2 (0.3)	42.7 (1.4)	-	434 (19.1)	3.9 (0.1)	27.4 (0.2)	58.4 (1.0)	0.08	340 (18.9)	3.1 (0.3)
	2	30.7 (0.4)	44.7 (1.7)	-	428 (22.4)	4.1 (0.2)	29.9 (0.4)	47.9 (1.3)	-	394 (21.7)	3.3 (0.1)	26.5 (0.4)	50.9 (1.0)	0.33	363 (21.9)	3.7 (0.2)
Average		27.1 (0.1)	42.2 (0.3)	53*	448 (4.5)	4.4 (0.1)	27.4 (0.1)	39.6 (0.3)	3.7*	442 (4.9)	3.9 (0.1)	23.7 (0.1)	56.6 (0.2)	21.8*	411 (4.8)	3.7 (0.1)

[1] 10-day interval period; [2] Ta air temperature (^◦^C); [3] RH air relative humidity (%); [4] R precipitation (mm); [5] R_S_ solar radiation (W m^−2^); [6] ET_OPM_ potential evapotranspiration (mm); *total accumulated values; Station A, agro-meteorological station, altitude 165 m, Cyprus; Station B, agro-meteorological station, altitude 330 m, Cyprus; Station C: agro-meteorological station, altitude 60 m, Italy

Higher air temperature values of 3.4 °C (station A) and 3.7 °C (station B) were recorded in Cyprus compared with station C located in Italy. The maximum air temperature observed in July was 39.8 °C (station A), 37.9 °C (station B), 38.7 °C (station C). The mean air temperature for a three-day representative period in July is illustrated in Fig. 2B. In July, during the second 10-day interval period, station C recorded the highest air temperature values.

In station A, the total recorded precipitation through the irrigation period was 53 mm. However, 43 mm of rain was recorded only on one day in June. In station B, the total precipitation was 3.7 mm, and in station C, it was 21.8 mm. Considering the number of rainy days in relation to the amount of precipitation, the effective rainfall (i.e., green water) during the irrigated period in the semi-arid Mediterranean region was considered negligible.

Over the study period, the mean ET_OPM_ values in the study regions ranged from 1.1 to 6.1 mm (maximum value of 6.7; station A). Significantly higher potential evapotranspiration (ET_OPM_) rates (p ≤ 0.05) were estimated for station A (12 and 18% increases compared to station B and station C respectively). The mean ET_OPM_ was 4.4 mm (station A), 3.9 mm (station B), and 3.7 mm (station C). Figure 2C shows the diurnal ET_OPM_ for a three-day period in July. It can be observed that in stations A and B, the ET_OPM_ values followed a similar daily trend. However, higher values of ET_OPM_ were recorded for station C during and after midday hours (Fig. 2D).

**Fig. 3:**
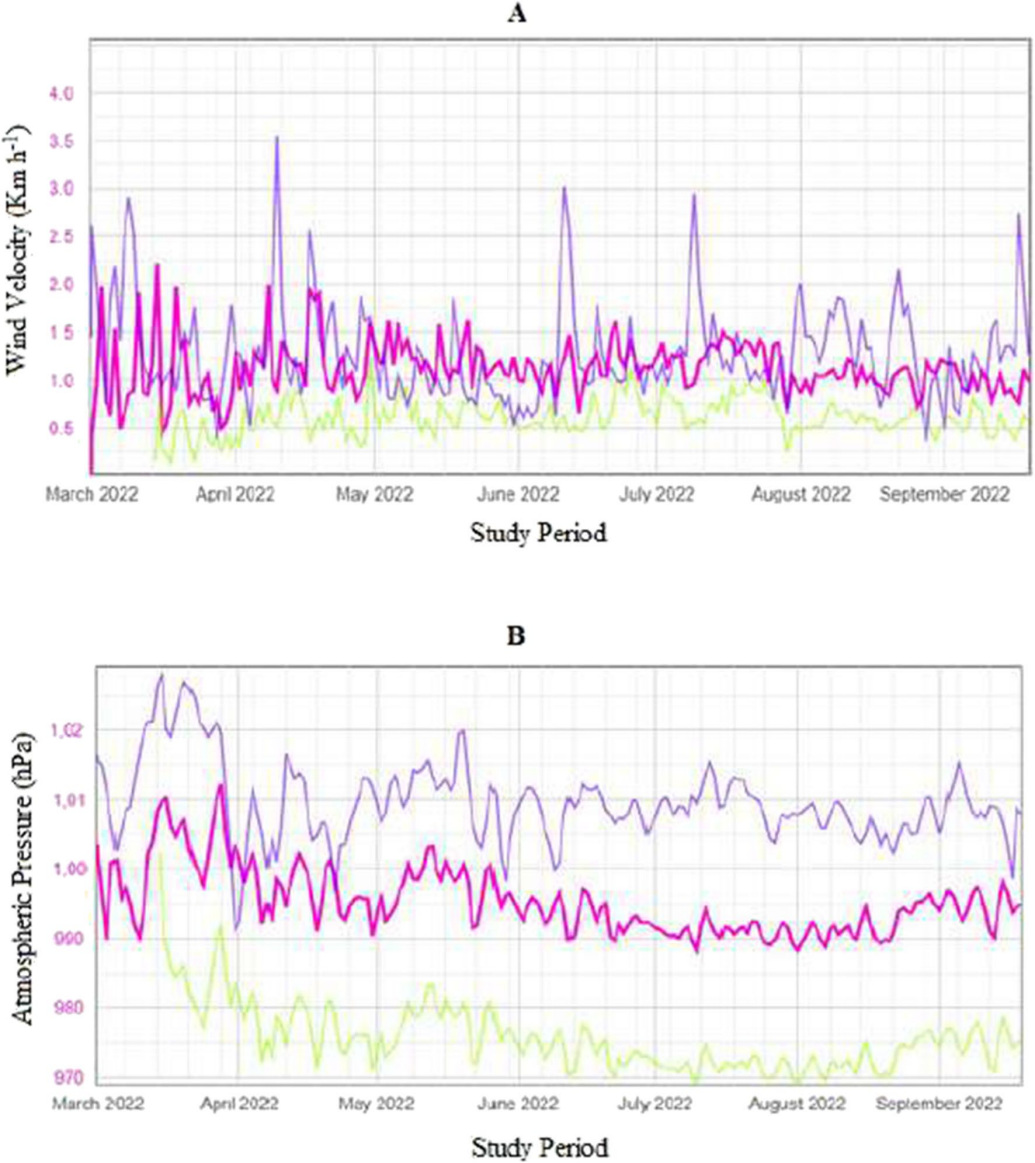
Mean daily wind velocities (A; Km h^−1^) and mean atmospheric pressure (B; hPa) over the study period; Red line (agro-meteorologigal station A; altitude 165 m; Cyprus); Green line (agro-meteorologigal station B; altitude 330 m; Cyprus); blue bars (agro-meteorologigal station C; altitude 60 m; Italy)

Figure 3 shows the mean wind velocities (Fig. 3A) and atmospheric pressure (Fig. 3B) during the measurement period in the three locations. It can be observed that the wind velocity was consistently lower at station B compared with stations A or C for the study period. These values show the expected differences caused by the crop surrounding the station. Considering the atmospheric pressure between Italy (station C) and Cyprus (stations A and B), higher values were observed on behalf of station C (Fig. 3B).

### Comparison of the Abtew method with the Penman–Monteith

Considering the net solar radiation values recorded in stations A and B and reference evapotranspiration based on the Penman–Monteith equation (ET_OPM_), we proceeded with the Abtew *k* coefficient calibration (Eq. 4). The calculated mean *k* coefficient values (*k* = λ ΕΤ_ΟPM_ / R_S_) were in the range of 1 to 1.46 (dimensionless), with an overall mean value of 1.22 (± 0.01) for stations A and B (Table 2). Over the study period (March to September), on 10-day interval measurement calculations, the result shows variations in *k* coefficient values within the same station and between different stations. The higher mean *k* values were calculated for both stations during September (avg. 1.46).

**Table 2:** Ten-day interval *k* coefficient values (dimensionless) in station A and B. Values in parenthesis represent (± standard error). Station A, agro-meteorological station, altitude 165 m, Cyprus; Station B, agro-meteorological station, altitude 330 m, Cyprus

	Station A				Station B			
Month		Mean	Minimum	Maximum	Mean	Minimum	Maximum	Mean
March	1	1.26 (0.11)	1.02	2.13	1.26 (0.11)	1.02	2.13	1.26(0.08)
	2	1.01(0.07)	0.66	1.53	1.10(0.13)	0.66	1.72	1.05(0.07)
	3	1.01(0.09)	0.21	1.26	1.00(0.11)	0.15	1.52	1.01(0.07)
April	1	1.27(0.02)	1.13	1.34	1.17(0.03)	1.01	1.36	1.22(0.02)
	2	1.24(0.09)	0.99	1.83	1.10(0.05)	0.90	1.47	1.17(0.05)
	3	1.21(0.04)	0.99	1.44	1.11(0.04)	0.95	1.35	1.16(0.03)
May	1	1.20(0.02)	1.11	1.32	1.14(0.02)	1.08	1.25	1.17(0.02)
	2	1.39(0.19)	1.14	3.05	1.11(0.02)	1.04	1.25	1.25(0.10)
	3	1.22(0.03)	1.11	1.41	1.16(0.02)	1.03	1.29	1.19(0.02)
June	1	1.15(0.04)	1.08	1.44	1.04(0.01)	0.98	1.08	1.09(0.02)
	2	1.18(0.03)	1.03	1.33	1.21(0.05)	1.04	1.55	1.19(0.03)
	3	1.25(0.02)	1.14	1.40	1.17(0.02)	1.08	1.28	1.21(0.02)
July	1	1.29(0.06)	1.17	1.83	1.15(0.02)	1.09	1.23	1.22(0.04)
	2	1.30(0.02)	1.18	1.36	1.19(0.02)	1.04	1.27	1.24(0.02)
	3	1.30(0.02)	1.17	1.39	1.22(0.02)	1.11	1.27	1.26(0.02)
August	1	1.28(0.02)	1.18	1.43	1.21(0.02)	1.17	1.35	1.24(0.02)
	2	1.37(0.06)	1.26	1.91	1.28(0.06)	1.17	1.76	1.33(0.04)
	3	1.36(0.03)	1.20	1.47	1.24(0.02)	1.18	1.37	1.30(0.02)
September	1	1.45(0.01)	1.41	1.53	1.28(0.03)	1.18	1.41	1.37(0.02)
	2	1.45(0.04)	1.38	1.73	1.46(0.07)	1.18	1.86	1.46(0.04)
Mean		1.26(0.02)	0.21	3.05	1.18(0.01)	0.15	2.13	1.22(0.01)

**Fig. 4:**
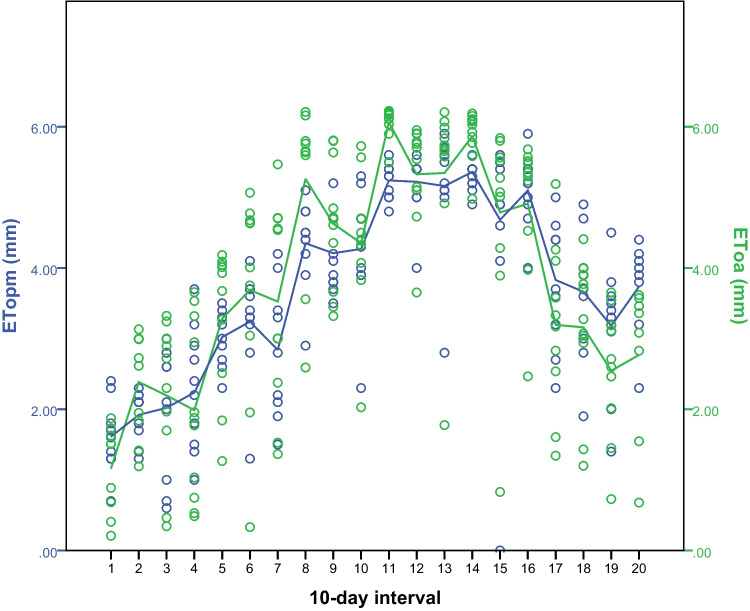
Mean daily variation of potential evapotranspiration estimated with the Penman–Monteith (ET_OPM_; blue line) and with the Abtew method (ET_OA_; green line) based on climatic parameters derived from station C (agro-meteorological station, altitude 60 m, Italy) over a 10-day interval period starting in March till September

The mean daily variation of potential evapotranspiration calculated based on the Penman-Monteith method (eq. 1) and the Abtew method (eq. 3, considering a new *k* coefficient value of 1.22) over a 10-day interval period (March to September), are shown in Fig. 4 (agro-meteorological station C, altitude 60 m, Italy). It can be observed that ET_OPM_ and ET_OA_ followed a similar trend over the study period. The slight over- and under-estimation of ET_OA_ could be explained due to variations of the “constant *k* coefficient”.

**Fig. 5:**
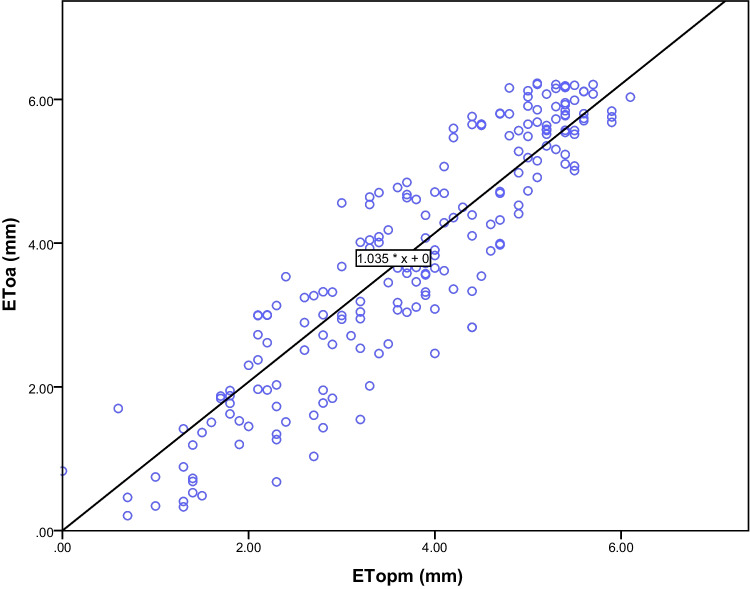
Calculated potential evapotranspiration values (Abtew equation; ET_OA_) plotted against potential evapotranspiration (Penman–Monteith equation; ET_OPM_)

Thus, using the calibrated *k* coefficient value (Cyprus) to measure ET_OA_ against ET_OPM_, in another location (Italy) with similar conditions, we developed a linear fitting of the measured parameters (Fig. 5). The r^2^ values (0.97) for many observations (n = 200) were statistically significant (P < 0.05), with a beta value of 0.98. The root mean square error (RMSE) was 0.61 mm d^−1^. The mean daily ET_OA_ was estimated at 3.75 (± 0.09) mm and ET_OPM_ at 3.84 (± 0.12) mm, which statistically (p ≤ 0.05) are considered identical.

### Comparison of the Jensen and Haise method with the Penman- Monteith

**Fig. 6:**
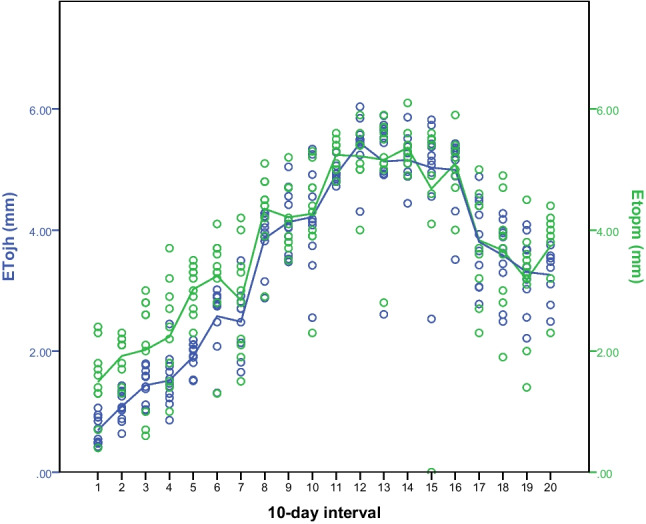
Mean daily variation of potential evapotranspiration estimated with the Penman–Monteith (ET_OPM_; green line) and with the Jense and Haise method (ET_OJH_; blue line) based on climatic parameters derived from station C (agro-meteorological station, altitude 60 m, Italy) over a 10-day interval period starting in March till September

The mean daily variation of potential evapotranspiration estimated with the Penman–Monteith (ET_OPM_; Eq. 1) and with the Jense and Haise method (ET_OJH_; Eq. 2) based on climatic parameters derived from station C is shown in Fig. 6. It can be observed that the two methods applied for potential evapotranspiration estimation showed a similar trend, with a slight underestimation during the first two months.

**Fig. 7:**
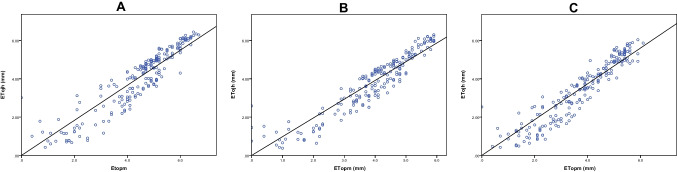
Calculated potential evapotranspiration values (Jensen and Hasie equation; ET_OJH_) plotted against potential evapotranspiration (Penman–Monteith equation; ET_OPM_); Station A, agro-meteorological station, altitude 165 m, Cyprus; Station B, agro-meteorological station, altitude 330 m, Cyprus; Station C, agro-meteorological station, altitude 60 m, Italy

A significant linear regression between ET_OJH_ and ET_OPM_ for different locations is shown in (Fig. 7), suggesting a good correspondence. The mean daily ET_OJH_ values were: 3.99 ± 0.11 mm (station A), 3.84 ± 0.10 mm (station B), and 3.48 ± 0.10 mm (station C), and accordingly, ET_OPM_ values were 4.41 (± 0.10 mm-station A), 3.97 (± 0.09 mm-station B), 3.48 (± 0.10 mm-station C). The r^2^ values for several observations (n = 200) were statistically significant (P < 0.05) accounting for 0.97 with a beta value of 0.98 for station A, 0.98 with a beta value of 0.97 for station B, and 0.97 with a beta value of 0.93 for station C.

### Crop evapotranspiration requirements based on the modified Abtew equation

Using the single crop coefficient method (Eq. 6) and the modified Abtew Eq. (7), the evapotranspiration requirements for olives and citrus were estimated for Cyprus (35.1264° N, 33.4299° E) on a ten-day interval basis (Table 3), considering historical extraterrestrial radiation (R_A_; Appendix Table 4) (Allen et al. 1998). The evapotranspiration requirements were also estimated for the same crops following the single crop coefficient method and the reference evapotranspiration (Penman–Monteith; Equation; Table 1). The results are illustrated in Table 3.

**Table 3:** Evapotranspiration requirements (mm) for olives and citrus estimated based on the modified Abtew equation and the FAO Penman–Monteith method

Month	10-day interval period	R_A_	ET_CA olives_	ET_CA citrus_	ET_CPM olives_	ET_CPM citrus_
March	1	26.46	13.37	14.40	11.05	12.75
	2	28.78	26.97	31.11	20.9	28.5
	3	31.17	27.19	31.37	22	30
April	1	33.41	18.97	31.62	17.1	28.5
	2	35.34	21.00	42.48	17.55	35.49
	3	37.03	21.13	42.72	21.15	42.77
May	1	38.44	21.23	42.92	19.8	40.04
	2	39.57	21.31	43.09	19.35	39.13
	3	40.44	23.31	47.14	24.75	50.05
June	1	41.00	23.31	47.14	20.7	41.86
	2	41.25	23.37	47.27	21.6	43.68
	3	41.23	23.37	47.27	24.75	50.05
July	1	40.94	21.40	43.28	24.75	50.05
	2	40.38	23.35	47.22	27.45	55.51
	3	39.49	23.24	46.99	25.2	50.96
August	1	38.31	23.15	46.81	22.5	45.5
	2	36.90	19.20	38.82	22.5	45.5
	3	35.14	19.20	38.82	19.8	40.04
September	1	33.14	27.38	38.33	29.25	40.95
	2	31.03	27.18	38.05	26.65	37.31
Sum			**448.62**	**806.84**	**438.80**	**808.64**

R_A,_ extraterrestrial radiation (MJ m^−2^ d^−1^); ET_CA_ crop evapotranspiration (mm) based on the modified Abtew equation; ET_CPM_ crop evapotranspiration (mm) based on the Penman–Monteith

The evapotranspiration requirements for olives and citrus, estimated using the modified Abtew equation with calibrated *k* coefficient and extraterrestrial radiation, closely matched the estimates obtained using the Penman–Monteith method (Table, 3) Furthermore, tresults on evapotranspiration for olive and citrus crops using a class A evaporation pan, along with the application of local crop coefficient values (K_C_ values), are in line with the findings of the current study (Christou et al. 2017; Nikolaou et al. 2020a, b).

## Discussion

The reliability of evapotranspiration models relying on limited climatic inputs is an important challenge in dry Mediterranean regions because the FAO-56 Penman–Monteith method cannot be applied in many situations due to the poor weather data collection facilities (Aschale et al. 2022). Thus, empirical equations of evapotranspiration based on easily measured parameters such as solar radiation that are adapted to the prevailing environmental conditions are considered useful tools, particularly in southern Europe, where irrigation scheduling is currently based on the experience of the growers (Incrocci et al. 2020). For example, incorporating these equations into irrigation schemes will enhance water productivity at the farm level. Furthermore, in the case of a large-scale irrigation policy, energy data could be retrieved based on satellite remote sensing or historical extraterrestrial radiation (Appendix Table 4).

Considering input climatic data and estimated potential evapotranspiration values (ET_OPM_), it was observed that ET_OPM_ in station B (agro-meteorological station, altitude 330 m, Cyprus) was lower than in station A (agro-meteorological station, altitude 165 m, Cyprus) and higher than in station C (agro-meteorological station, altitude 60 m, Italy). In this context, many researchers have investigated the effect of microclimate on potential evapotranspiration. They concluded that solar radiation has the largest impact on the reference evapotranspiration values, followed by the air temperature, the saturation vapour pressure deficit, and lastly, the wind speed (Maček et al. 2018). Considering stations A and B (which are both located in Cyprus), solar radiation and air temperature values were higher for station A, thereby resulting in higher evapotranspiration values. However, the higher wind speed that was observed at station C was not able to explain the lower evapotranspiration rate compared with station B. It is clear from the results that the effect of solar radiation was more intense on potential evapotranspiration than the air temperature values or wind speed, as may be expected. In addition, higher air humidity values in station C could also be associated with lower evapotranspiration rates. Considering the atmospheric pressure between Italy (station C) and Cyprus (stations A and B), higher values were observed on behalf of station C (Fig. 3). A distinct relationship between pressure and evaporative values is observed; thereby, at higher pressures, lower evaporation is expected.

It has been shown that the radiation-based methods tested have similar performance standards to the PM method in semi-arid (type BSh) and hot Mediterranean (type Csa) climatic regions, and they can estimate reference evapotranspiration with high accuracy once the equation coefficients are adjusted to the local environmental conditions. Those *k* values of the calibrated Abtew equation obtained under our experimental conditions were much higher than those initially proposed and used by many researchers or even those found in the literature. However, according to Samaras et al. (2014), the *k* coefficient in Abrtew’s model for evapotranspiration calculation is also related to the air Tmax of the region. Indeed, this is justified in our case by the lower values of the *k* coefficient for station B compared to station A, where the higher values of air Tmax were recorded, and probably explains the higher values of *k* found in our experiments.

Xu and Singh 2000, working with several radiation-based equations for determining evaporation, indicated that the original constant value of the Abtew equation (*k* = 0.53) agreed most closely with pan evaporation in Switzerland, compared with other models without recalibration (i.e., Hargreaves, Makkink, Priestley and Taylor and Turc). In contrast, Shirmohammadi-Aliakbarkhani and Saberali (2020) suggested that the Abtew equation based on a coefficient *k* = 0.53 might be unreliable for evapotranspiration estimation in arid regions of Iran. In West Africa, the calibrated Abtew method showed the best performance among nine tested ETo equations (Djaman et al. 2017). Similarly, (Samaras et al. 2014) reported that the Abtew model (calibrated and validated) showed the best overall performance to the data from all available climate stations under different Mediterranean climates in central Greece. In our case, with a regression slope of almost unity and a very high correlation coefficient between ET_OPM_ and ET_OA_, the calibrated equation (*k* = 1.22) showed good performance and can be used for potential evapotranspiration estimation in semi-arid and hot Mediterranean climates, accounting for cases where solar radiation is the only available climatic parameter. In addition, the given modified Abtew equation using historical extraterrestrial solar radiation data (Appendix Table 4), can be a useful tool for decision-making when it comes to predicting water deliveries in advance.

The results also indicate that the Jensen and Haise model provides good estimates of ET_O_. Therefore, we may also consider this method for estimating potential evapotranspiration in semi-arid and hot Mediterranean climate regions. Indeed, among the radiation-based methods, the Jensen − Haise method was the only one that exhibited consistent results in some regions (Shirmohammadi-Aliakbarkhani and Saberali 2020). Although other authors (Ahmadi and Javanbakht 2020) have suggested that the Jensen and Haise method tends to overestimate potential evapotranspiration in some cases. This can be explained, as cited by Yang et al. (2021), due to the differences in selected models, variables used to validate evaporation, evaluation criteria, or evaluation scales, different studies have reached different conclusions regarding the best radiation-based models.

Another point for consideration is the latent heat of vaporization (i.e., the amount of energy required to change liquid water into water vapor) in relation to the surrounding agro-meteorological station. The energy is provided by various sources, including the atmosphere above, the soil surrounding, and the inflowing water (Cox 1999). Comparing station A with station B, there was a great difference regarding the surrounding crop cultivation, thereby affecting the micro-environmental conditions. Station A was located in a field with aromatic plants (crop evapotranspiration requirements of 250–350 mm) compared with olive crops (crop evapotranspiration requirements of 450 mm).

Considering climatic data limitations and current results, a machine learning model based on the solar energy fluxes at the surface could be used as a simple and robust approach to controlling irrigation frequency. Given the daily or weekly amount of water needed by the crop and calculated with the above-described methods, the frequency and duration of each irrigation shift can be automatically tuned, allowing the water to flow at root level while minimizing surface evaporation and stratum percolation. To achieve this aim, soil moisture sensors can be installed in the field to provide feedback on the water flow after the irrigation shift, to adapt the frequency and duration, and to ensure the proper amount of water is returned to the crop.

## Conclusion

In semi-arid and hot Mediterranean climate regions, the Penman–Monteith method was compared to two empirical solar radiation methods (Abtew and Jensen and Haise) to compute daily reference evapotranspiration values. The findings demonstrated that the radiation-based methods were precise in estimating the potential evapotranspiration in the designated study areas. The Abtew method, in contrast to Jensen and Haise, is known for its simplicity when calculating reference evapotranspiration, provided that the empirical constant is calibrated. As a result, the Abtew method can be suggested as a novel water management approach for scheduling irrigation using smart irrigation controllers connected to a basic solar radiation sensor. Furthermore, by adjusting the Abtew equation to account for extraterrestrial radiation, effective irrigation requirements can be determined. Considering climatic data limitations in many locations, simple solar-radiation-based equations could be used for proper irrigation scheduling.

## Appendix 1

Please see Table 4.

**Table 4:** Mean ten-day interval extraterrestrial solar radiation values (R_A_; MJ m^−2^ d^−1^) for latitudes of Mediterranean countries (Kittas 1990)

		Latitude							
Day-interval	32̊		34̊	36̊	38̊	40̊	42̊	44̊	46̊
January									
1	18.84		17.63	16.41	15.19	13.97	12.74	11.52	10.31
2	19.72		18.53	17.33	16.11	14.90	13.68	12.46	11.24
3	21.05		19.89	18.71	17.52	16.32	15.12	13.90	12.69
February									
1	22.73		21.61	20.48	19.32	18.16	16.97	15.78	14.58
2	24.59		23.53	22.45	21.35	20.23	19.09	17.93	16.75
3	26.40		25.42	22.40	23.36	22.30	21.21	20.10	18.96
March									
1	28.29		27.39	26.46	25.50	24.51	23.49	22.44	21.36
2	30.40		29.61	28.78	27.92	27.03	26.10	25.14	24.16
3	32.53		31.17	31.17	30.43	29.66	28.85	28.00	27.13
April									
1	34.49		33.97	33.41	32.81	32.17	31.49	30.77	30.02
2	36.15		35.77	35.34	34.87	34.37	33.82	33.24	32.62
3	37.57		37.32	37.03	36.69	36.32	35.91	35.46	34.97
May									
1	38.73		38.61	38.44	38.23	37.98	37.70	37.38	37.02
2	39.64		39.62	39.57	39.47	39.33	39.16	38.96	38.72
3	40.33		40.40	40.44	40.44	40.40	40.32	40.22	40.08
June									
1	40.77		40.90	41.00	41.07	41.10	41.09	41.06	40.99
2	40.95		41.12	41.25	41.35	41.42	41.45	41.45	41.42
3	40.92		41.10	41.23	41.33	41.40	41.43	41.44	41.41
July									
1	40.69		40.83	40.94	41.01	41.05	41.05	41.02	40.97
2	40.24		40.33	40.38	40.39	40.37	40.31	40.22	40.10
3	39.53		39.53	39.49	39.41	39.30	39.15	38.97	38.75
August									
1	38.57		38.46	38.31	38.12	37.79	37.63	37.32	36.99
2	37.40		37.17	36.90	36.59	36.24	35.85	35.42	34.96
3	35.92		35.55	35.14	34.69	34.21	33.68	33.12	32.51
September									
1	34.19		33.68	33.14	32.55	31.92	31.26	30.56	29.82
2	32.35		31.71	31.03	30.31	29.56	28.77	27.95	27.09
3	30.36		29.60	28.80	27.96	27.10	26.20	25.26	26.30
October									
1	28.30		27.41	26.52	25.58	24.61	23.61	22.59	21.54
2	26.23		25.26	24.27	23.24	22.19	21.11	20.02	18.50
3	24.17		23.11	22.04	20.94	19.82	18.69	17.54	16.37
November									
1	22.29		21.18	20.04	18.89	17.73	16.55	15.36	14.17
2	20.77		19.61	18.44	17.26	16.06	14.86	13.65	12.45
3	19.58		18.39	17.19	15.98	14.77	13.55	12.34	11.13
December									
1	18.75		17.55	16.33	15.11	13.89	12.67	11.45	10.24
2	18.34		17.12	15.90	14.67	13.45	12.22	11.00	9.80
3	18.36		17.14	15.92	14.69	13.46	12.24	11.02	9.81
